# Development and Pilot of a Checklist for Management of Acute Liver Failure in the Intensive Care Unit

**DOI:** 10.1371/journal.pone.0155500

**Published:** 2016-05-13

**Authors:** Oren K. Fix, Iris Liou, Constantine J. Karvellas, Daniel R. Ganger, Kimberly A. Forde, Ram M. Subramanian, Alice Boylan, James Hanje, R. Todd Stravitz, William M. Lee, Acute Liver Failure Study Group

**Affiliations:** 1 Swedish Medical Center, Organ Transplant Program, Seattle, Washington, United States of America; 2 University of Washington, Department of Medicine, Seattle, Washington, United States of America; 3 University of Alberta, Department of Medicine, Edmonton, Alberta, Canada; 4 Northwestern University, Department of Medicine, Chicago, Illinois, United States of America; 5 University of Pennsylvania, Department of Medicine, Philadelphia, Pennsylvania, United States of America; 6 Emory University, Department of Medicine, Atlanta, Georgia, United States of America; 7 Medical University of South Carolina, Department of Medicine, Charleston, South Carolina, United States of America; 8 The Ohio State University, Department of Medicine, Columbus, Ohio, United States of America; 9 Virginia Commonwealth University, Department of Medicine, Richmond, Virginia, United States of America; 10 University of Texas Southwestern, Department of Medicine, Dallas, Texas, United States of America; Chiba University, Graduate School of Medicine, JAPAN

## Abstract

**Introduction:**

Acute liver failure (ALF) is an ideal condition for use of a checklist. Our aims were to develop a checklist for the management of ALF in the intensive care unit (ICU) and assess the usability of the checklist among multiple providers.

**Methods:**

The initial checklist was developed from published guidelines and expert opinion. The checklist underwent pilot testing at 11 academic liver transplant centers in the US and Canada. An anonymous, written survey was used to assess the usability and quality of the checklist. Written comments were used to improve the checklist following the pilot testing period.

**Results:**

We received 81 surveys involving the management of 116 patients during the pilot testing period. The overall quality of the checklist was judged to be above average to excellent by 94% of users. On a 5-point Likert scale, the majority of survey respondents agreed or agreed strongly with the following checklist characteristics: the checklist was easy to read (99% agreed/agreed strongly), easy to use (97%), items are categorized logically (98%), time to complete the checklist did not interfere with delivery of appropriate and safe patient care (94%) and was not excessively burdensome (92%), the checklist allowed the user the freedom to use his or her clinical judgment (80%), it is a useful tool in the management of acute liver failure (98%). Web-based and mobile apps were developed for use of the checklist at the point of care.

**Conclusion:**

The checklist for the management of ALF in the ICU was shown in this pilot study to be easy to use, helpful and accepted by a wide variety of practitioners at multiple sites in the US and Canada.

## Introduction

### Acute liver failure management in the intensive care unit

Acute liver failure (ALF) is caused by the sudden loss of liver function and defined by coagulopathy and encephalopathy in a patient without known pre-existing liver disease [1]. It is a complex medical condition involving critically ill patients with a high mortality, and requires an intensive care unit (ICU) setting and multidisciplinary team of providers to ensure the best possible outcome. The clinical course and complications from this syndrome are variable depending on the etiology of the liver failure, timing of presentation to medical care and inconsistent medical practices. ALF is a rare condition with an estimated incidence of 2,000 cases per year in the United States [2]. There are few controlled trials evaluating specific treatments for ALF. In spite of this, the management of ALF has advanced over the decades, best practices have evolved and outcomes have improved [3]. The advancement of electronic medical record systems and growing use of computerized physician order entry presents an opportunity for a standardized approach to optimize the management of ALF.

### Checklists in medicine

Checklists are becoming more prevalent in medicine, particularly in the ICU. They have been shown to decrease medical errors, improve standards of patient care and improve adherence to best practices, particularly during complex tasks [4, 5]. Well-known examples of checklists in medicine include the Catheter-Related Blood Stream Infection checklist developed at Johns Hopkins, which decreased the incidence of these infections from 11.3 to 0 per 1000 catheter-days [6]. The Surgical Safety Checklist, pioneered by Atul Gawande and the World Health Organization, reduced the risk of perioperative death from 1.5% to 0.8% and the risk of inpatient complications from 11% to 7% [7]. The success of these checklists derives in part from defined interventions and outcomes of interest.

We hypothesized that ALF is an ideal condition for use of a checklist because it requires a multidisciplinary team of providers to analyze and manage a highly complex condition in a demanding and stressful ICU setting. Our aims were to develop a checklist for the management of ALF in the ICU and assess the usability of the checklist among multiple types of providers in order to standardize and improve management of ALF in the ICU.

## Methods

The initial checklist was developed from December 2010 to March 2012. We used published guidelines [8, 9] and expert opinion (selected principal investigators in the Acute Liver Failure Study Group), recognizing that few randomized controlled trials exist to guide management of ALF. Items with insufficient or controversial data were included when there was consensus that the recommendation was beneficial. Initial drafts were reviewed by the experts and refined iteratively through consensus.

The checklist underwent pilot testing from May 2012 to April 2013 at 11 academic liver transplant centers in the US and Canada (Table 1). Each site was led by an experienced transplant hepatologist. Five sites were not originally among the ALFSG network, of which 4 sites later joined the group. Three of these 4 new sites had investigators who were dual-certified in both transplant hepatology and critical care. We developed an online instructional video (http://alfchecklist.com/video) to demonstrate how to use the checklist.

**Table 1:** Checklist pilot sites.

University of California San Francisco, California, USA
University of Texas Southwestern, Dallas, Texas, USA
Medical University of South Carolina, Charleston, South Carolina, USA
Northwestern University, Chicago, Illinois, USA
University of Washington, Seattle, Washington, USA
University of Pennsylvania, Philadelphia, Pennsylvania, USA
University of Colorado, Denver, Colorado, USA
Yale University, New Haven, Connecticut, USA
University of Alberta, Edmonton, Alberta, Canada
The Ohio State University, Columbus, Ohio, USA
Emory University, Atlanta, Georgia, USA

**Fig 1:**
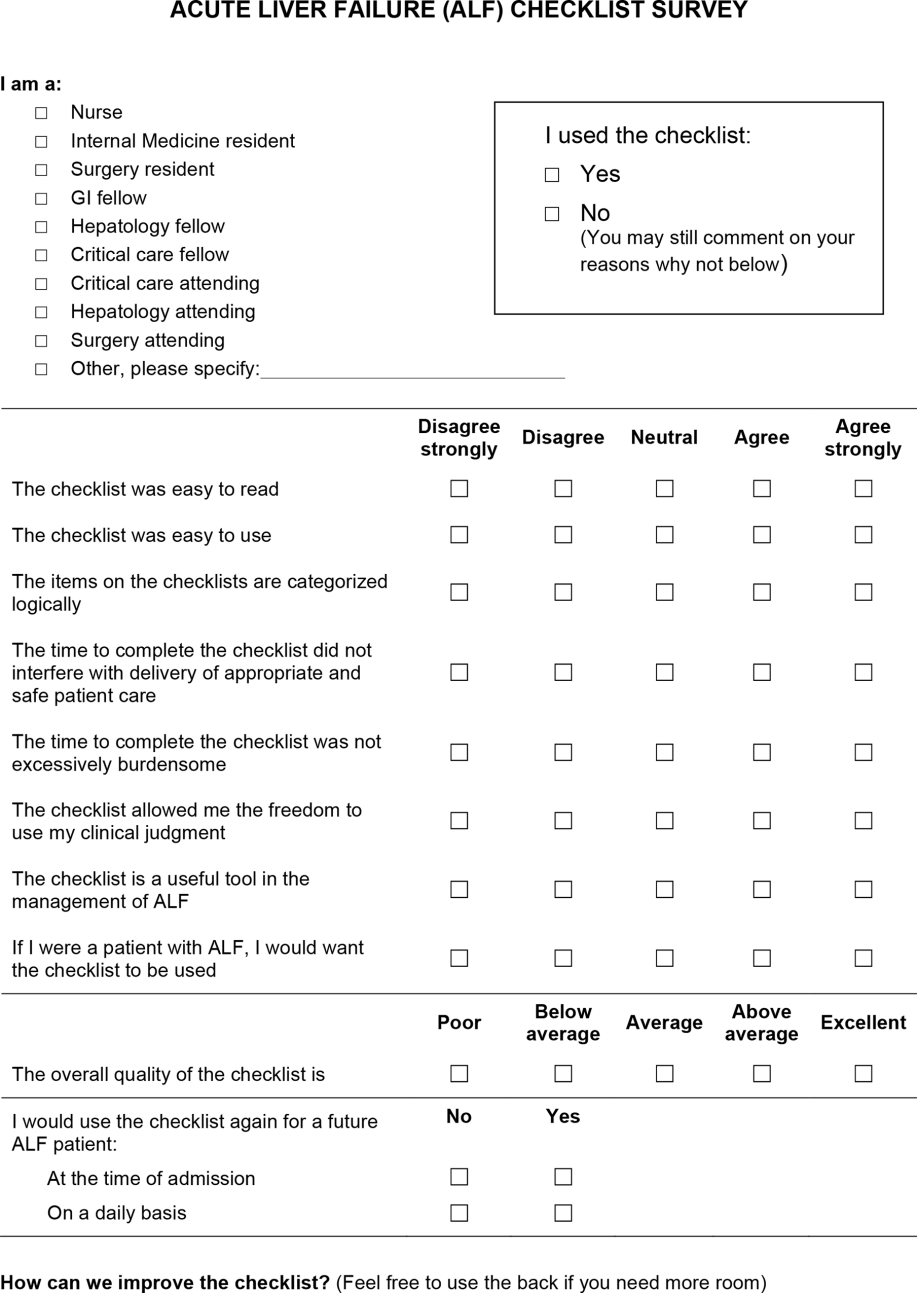
Checklist pilot survey.

An anonymous, written survey form was used to assess the usability and quality of the checklist (Fig 1). Health care providers were the research subjects. The study was explained to the provider and an information sheet was provided. Verbal consent was obtained and documentation of consent was assumed by the research subject’s completion of the survey. We surveyed multiple checklist users per patient but only a single survey was administered to each research subject for the duration of the pilot study. Written comments were used to improve the checklist following the pilot testing period.

Institutional review boards at each of the following sites approved the study: University of California, San Francisco, California, USA; University of Texas Southwestern, Dallas, Texas, USA; Medical University of South Carolina, Charleston, South Carolina, USA; Northwestern University, Chicago, Illinois, USA; University of Washington, Seattle, Washington, USA; University of Pennsylvania, Philadelphia, Pennsylvania, USA; University of Colorado, Denver, Colorado, USA; Yale University, New Haven, Connecticut, USA; University of Alberta, Edmonton, Alberta, Canada (Research Ethics Board); The Ohio State University, Columbus, Ohio, USA; Emory University, Atlanta, Georgia, USA.

## Results

### Initial checklist design

The initial checklist design included 3 distinct sections on 2 pages. The checklist was also separated into sections to be reviewed on admission to the ICU and on a daily basis. The first section contained a list of best practices recommended for every ALF patient in the ICU. Recognizing that etiology is the most important determinant of prognosis, the first page contained a table of etiologies, followed by items that should be completed to identify the cause of ALF. Some of these items were recommended for every ALF patient admitted to the ICU regardless of the presumed etiology. The second page was organized by organ system, containing questions for each system designed to prompt action according to the patient’s status.

### Pilot testing

**Fig 2:**
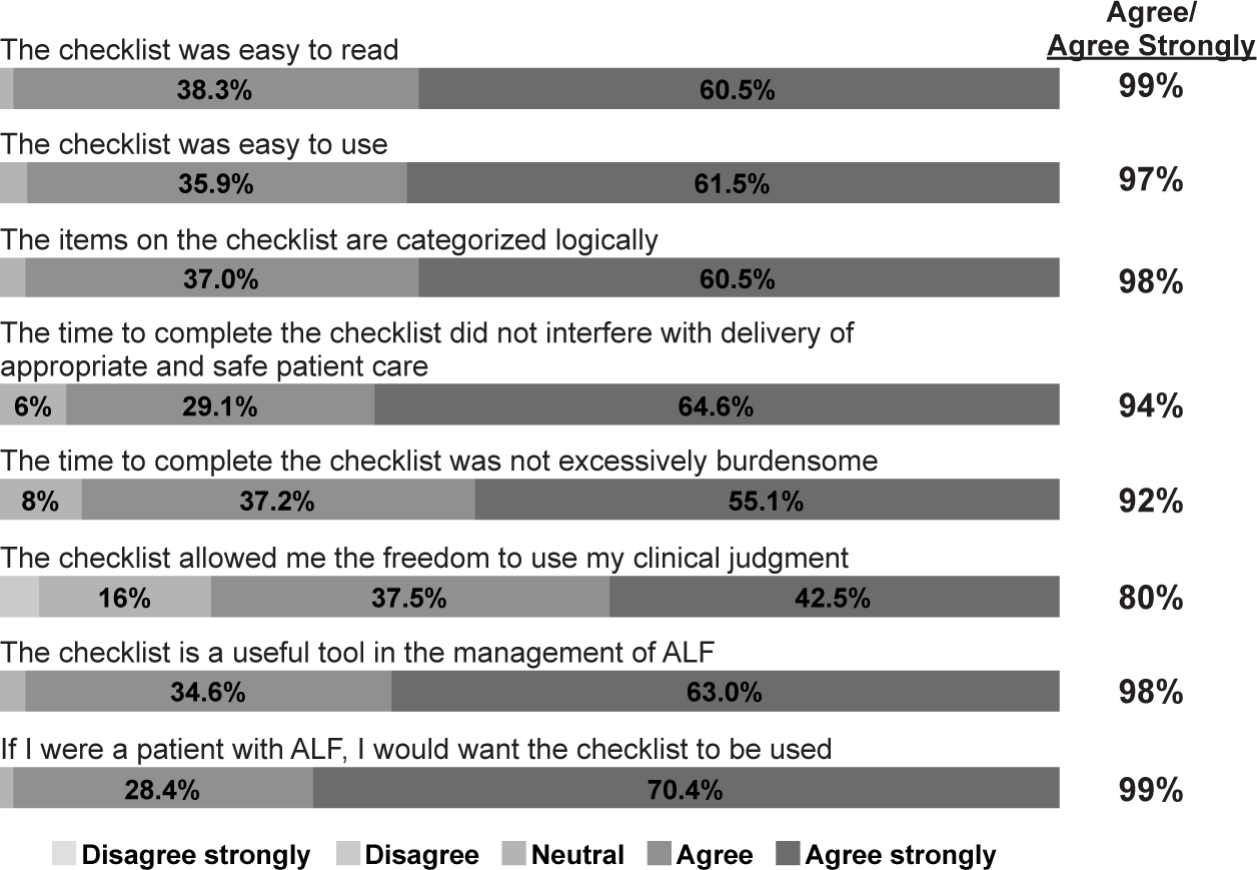
Pilot survey results.

We received 81 surveys from the 11 pilot sites (Table 1) involving the management of 116 ALF patients during the pilot testing period. A variety of different users completed the surveys, including physicians and non-physicians, faculty and trainees (Table 2). Among checklist users surveyed, 99% agreed or agreed strongly that the checklist was easy to read, 97% agreed or agreed strongly that it was easy to use, 98% agreed that the items on the checklist are categorized logically, 94% agreed that the time to complete the checklist did not interfere with delivery of appropriate and safe patient care, 92% agreed that the time to complete the checklist was not excessively burdensome. 80% agreed or agreed strongly that the checklist allowed the user the freedom to use his or her clinical judgment, 98% agreed the checklist is a useful tool in the management of ALF, and 99% agreed or agreed strongly that they would want the checklist to be used if they were a patient with ALF (Fig 2). All checklist users stated they would use the checklist again at the time of admission and 85% would use it on a daily basis. The overall quality of the checklist was judged to be above average to excellent by 94% of users.

**Table 2:** Checklist users.

**Staff Physicians**		**19**
	Critical care	15
	Hepatology	4
**Trainees**		**48**
	Gastroenterology fellow	10
	Critical care fellow	4
	Hepatology fellow	7
	Transplant surgery fellow	1
	Internal medicine resident	21
	Surgery resident	2
	Emergency resident	1
	Anesthesia resident	1
	Medical student (4^th^ year)	1
**Other**		**14**
	Nurse	10
	Nurse Practitioner	3
	Critical care pharmacist	1

Subgroup analysis of the item that received the lowest rating (80% agreed or agreed strongly that the checklist allowed the user the freedom to use his or her clinical judgment) showed that non-physicians were more likely than physicians to disagree or respond neutrally that the checklist allowed for clinical judgment (29% vs. 18%), while more senior staff physicians disagreed with this statement compared to trainees (30% vs 16%).

**Fig 3:**
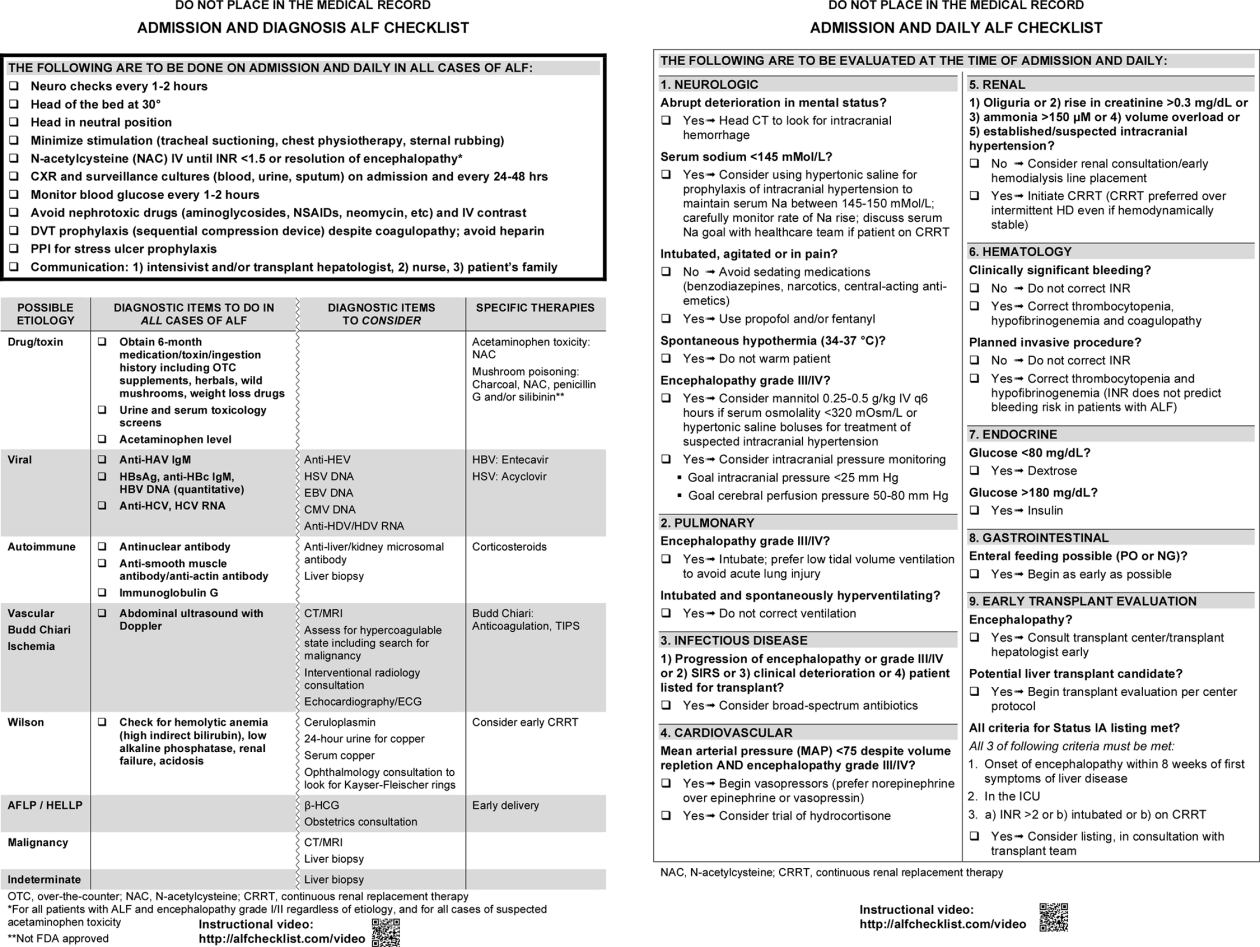
Final checklist.

Data obtained from pilot testing, including quantitative feedback and written comments, were used to revise the checklist (Table 3). The final checklist is shown in Fig 3.

**Table 3:** Examples of changes made to the checklist as a result of pilot testing.

Increased mean arterial pressure goal from 65 to 75 mm Hg for patients with encephalopathy grade III/IV
Clearly differentiated prophylaxis of intracranial hypertension from treatment
Specified that hypertonic saline should be used for prophylaxis of intracranial hypertension rather than treatment
Removed specific references to concentration and mode of delivery of hypertonic saline in order to acknowledge different practices across centers
More clearly stated the preference for continuous renal replacement therapy over intermittent hemodialysis when initiating renal replacement therapy
Separated “clinically significant bleeding” from “planned invasive procedure” to avoid promoting unnecessary correction of the international normalized ratio for invasive procedures
Increased the glucose upper limit from 150 to 180 mg/dL

## Discussion

Management of ALF in the ICU is not an exact science, with disparate practices at each center even within the cohesive and long-standing network of centers comprising the Acute Liver Failure Study Group. The lack of randomized controlled trials and established clinical endpoints, other than transplant-free survival, further complicates progress toward a standardized approach. Therefore, a checklist for the management of ALF in the ICU would seem an appropriate step to provide a synthesis of expert opinion, consensus and iteration guided by both quantitative and qualitative feedback through pilot testing.

Our pilot testing revealed that the preliminary checklist was a helpful tool for the management of ALF in the ICU, and was considered to be organized logically and easy to use. The vast majority of surveyed users found the checklist to be above average to excellent on a 5-point Likert scale, would use the checklist on a daily basis for future ALF patients and would want the checklist to be used if they themselves were a patient with ALF.

One of the goals of the checklist is to standardize and optimize ICU care for this rare syndrome. Given the highly complex management required by these critically ill patients who often experience rapid fluctuations in clinical status, this checklist can serve as a useful tool and a reminder of recommended practices. The checklist recommendations can be easily translated to physician order sets adapted to local institutional practices.

There are limitations to using checklists in medicine. Overuse of checklists can overburden clinicians, unnecessarily complicate tasks, increase complexity and reduce efficiency [5]. Some of the survey items we included were meant to explore whether our checklist suffered from these limitations, but we found high levels of agreement indicating the checklist did not interfere with delivery of appropriate and safe patient care, was not excessively burdensome and generally allowed providers the freedom to use their clinical judgment.

Five respondents specifically commented that the checklist is not sufficiently instructive and that the reasons for some recommendations are not elaborated (e.g., “Better rationale regarding explanations of course of actions”, “Include a section on managing metabolic acidosis”, “Define grade III/IV encephalopathy”). We designed the checklist, not as a teaching tool, but rather as a reminder of critical management steps. The ideal checklist user is the experienced provider who is comfortable with the management of ALF in the ICU rather than the novice who is seeking an algorithmic approach. In a limited way, however, some users, particularly trainees, found the checklist to be useful as an educational resource.

Another criticism of the checklist was that some recommendations do not correspond with institution-specific practices (e.g., “I would like to see the checklist be more center specific”, “Extensive mention of intracranial pressure monitoring, which I haven’t seen used often”). One of our aims was to standardize the management of ALF across centers. While we attempted as best as possible to create a checklist that was generalizable and respected institution-specific nuances, we recognized that it is impractical to design a checklist that incorporates all such practices.

The item on the survey that received the lowest rating of 80% (the checklist allowed the user the freedom to use his or her clinical judgment)–still a very favorable rating–suggested qualitatively that some users felt the checklist did in some ways interfere with freedom to use clinical judgment. Based on written comments, one reason for the lower ratings for this item was due to the table of etiologies on the first page of the checklist. In this table, the checklist directs the user to order a number of tests regardless of possible etiology, which includes checking viral and autoimmune serologies. Some users criticized this recommendation as being unnecessary when the diagnosis is known, for example in some cases of acetaminophen overdose (e.g., “No need for all labs in cases where acetaminophen overdose clear”, “An autoimmune panel may not be necessary in a case of clear acetaminophen toxicity”). After discussing this issue among the experts, we chose to leave this as a recommended part of the management of a patient with ALF, recognizing how common some of these etiologies are and the possibility for multiple etiologies to exist simultaneously and perhaps influence prognosis. As one of our goals was to standardize and improve management of ALF based on published guidelines and expert opinion, some restriction in the freedom to deviate from the checklist may be considered a desirable feature.

All checklist users were willing to use the checklist at the time of admission and a high proportion reported they would use it on a daily basis. The checklist may be more critical at the time of admission when an initial management plan is formulated and the majority of orders are written; however, the daily assessment is still crucial to the management of ALF patients who often progress rapidly. Promoting use of a point-of-care tool such as a Web-based or mobile app may simplify the daily assessment and encourage more frequent use of the checklist.

### Point-of-care apps

Web-based and mobile apps were developed to facilitate future use of the finalized version of the checklist at the point of care. The Web-based app, which is optimized for use with mobile devices, contains tooltips to explain some of the checklist items and also contains links to additional resources and background information for the management recommendations. The Web-based application can be found at http://alfchecklist.com and the iOS mobile app is available on the Apple^**®**^ App Store.

### Future directions

We plan to revise this checklist as new data and best practices on the management of ALF in the ICU emerge. Extension of use of this checklist to non-university-based liver transplant centers and community hospitals without liver transplant services could be explored to broaden its utility. Creating a condensed version of the checklist for the emergency department setting is another option to expand its function. The clinical impact of the checklist was not evaluated in this study; therefore, it will be important to assess use of the checklist on clinically relevant outcomes in a future study.

## Conclusion

The Acute Liver Failure Study Group checklist for the management of acute liver failure in the intensive care unit was shown in this pilot study to be easy to use, helpful and accepted by a wide variety of practitioners at multiple sites in the US and Canada. Future studies will need to determine the impact of the checklist on management of acute liver failure.

## Supporting Information

S1 File Minimal Data Set. (XLSX)
